# *In vivo* partial cellular reprogramming enhances liver plasticity and regeneration

**DOI:** 10.1016/j.celrep.2022.110730

**Published:** 2022-04-26

**Authors:** Tomoaki Hishida, Mako Yamamoto, Yuriko Hishida-Nozaki, Changwei Shao, Ling Huang, Chao Wang, Kensaku Shojima, Yuan Xue, Yuqing Hang, Maxim Shokhirev, Sebastian Memczak, Sanjeeb Kumar Sahu, Fumiyuki Hatanaka, Ruben Rabadan Ros, Matthew B. Maxwell, Jasmine Chavez, Yanjiao Shao, Hsin-Kai Liao, Paloma Martinez-Redondo, Isabel Guillen-Guillen, Reyna Hernandez-Benitez, Concepcion Rodriguez Esteban, Jing Qu, Michael C. Holmes, Fei Yi, Raymond D. Hickey, Pedro Guillen Garcia, Estrella Nuñez Delicado, Antoni Castells, Josep M. Campistol, Yang Yu, Diana C. Hargreaves, Akihiro Asai, Pradeep Reddy, Guang-Hui Liu, Juan Carlos Izpisua Belmonte

**Affiliations:** 1 Gene Expression Laboratory, Salk Institute for Biological Studies, 10010 North Torrey Pines Road, La Jolla, CA 92037, USA; 2 Razavi Newman Integrative Genomics and Bioinformatics Core, Salk Institute for Biological Studies, 10010 North Torrey Pines Road, La Jolla, CA 92037, USA; 3 Altos Labs, 5510 Morehouse Drive, San Diego, CA 92121, USA; 4 Molecular and Cell Biology Laboratory, Salk Institute for Biological Studies, 10010 North Torrey Pines Road, La Jolla, CA 92037, USA; 5 Division of Biological Sciences, UCSD, La Jolla, CA 92037, USA; 6 State Key Laboratory of Stem Cell and Reproductive Biology, Institute of Zoology, Chinese Academy of Sciences, Beijing 100101, China; 7 Ambys Medicines, 131 Oyster Point Boulevard, Suite 200, South San Francisco, CA 94080, USA; 8 Clinica CEMTRO, 28035 Madrid, Spain; 9 Universidad Católica San Antonio de Murcia (UCAM), Campus de los Jerónimos, N° 135 12, 30107 Guadalupe, Spain; 10 Hospital Clinic of Barcelona, Carrer Villarroel, 170, 08036 Barcelona, Spain; 11 Division of Gastroenterology, Hepatology and Nutrition, Cincinnati Children’s Hospital Medical Center, Cincinnati, OH 45229, USA; 12 Department of Pediatrics, College of Medicine, University of Cincinnati, Cincinnati, OH 45229, USA; 13 State Key Laboratory of Membrane Biology, Institute of Zoology, Chinese Academy of Sciences, Beijing 100101, China; 14 Laboratory of Biological Chemistry, School of Pharmaceutical Sciences, Wakayama Medical University, 25-1 Shitibancho, Wakayama, Wakayama 640-8156, Japan; 15 These authors contributed equally; 16 Lead contact

## Abstract

Mammals have limited regenerative capacity, whereas some vertebrates, like fish and salamanders, are able to regenerate their organs efficiently. The regeneration in these species depends on cell dedifferentiation followed by proliferation. We generate a mouse model that enables the inducible expression of the four Yamanaka factors (Oct-3/4, Sox2, Klf4, and c-Myc, or 4F) specifically in hepatocytes. Transient *in vivo* 4F expression induces partial reprogramming of adult hepatocytes to a progenitor state and concomitantly increases cell proliferation. This is indicated by reduced expression of differentiated hepatic-lineage markers, an increase in markers of proliferation and chromatin modifiers, global changes in DNA accessibility, and an acquisition of liver stem and progenitor cell markers. Functionally, short-term expression of 4F enhances liver regenerative capacity through topoisomerase2-mediated partial reprogramming. Our results reveal that liver-specific 4F expression *in vivo* induces cellular plasticity and counteracts liver failure, suggesting that partial reprogramming may represent an avenue for enhancing tissue regeneration.

## In brief

In regenerating animals, such as fish and salamanders, dedifferentiation followed by proliferation contributes to tissue regeneration. Hishida et al. show that hepatocyte-specific cellular reprogramming induces cell proliferation and dedifferentiation in the liver and enhances liver regenerative capacity through topoisomerase2-mediated partial reprogramming.

## Graphical abstract

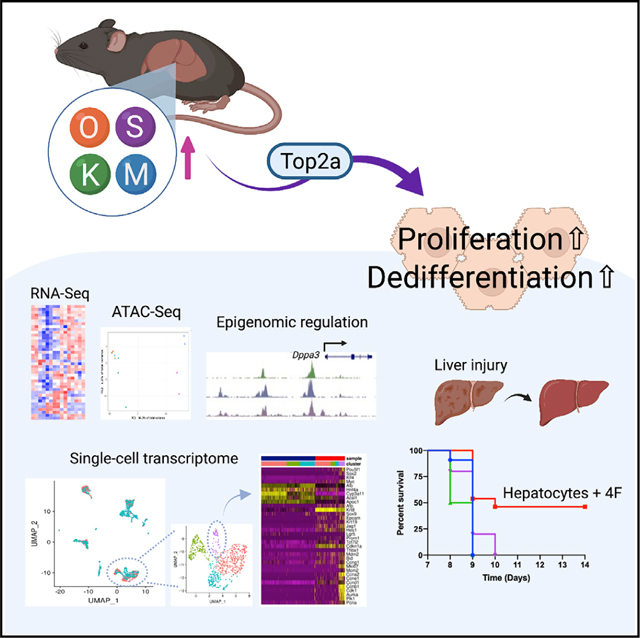

## INTRODUCTION

Mammals lack the regenerative capacity exhibited by some vertebrates, such as fish and salamanders (Simon, 2012). In these regenerating animals, it has been shown that dedifferentiation followed by proliferation contributes to tissue regeneration (Jopling et al., 2010; Wang and Simon, 2016). The scarcity of dedifferentiation in mammal tissues (Rinkevich et al., 2011) may be the reason they cannot regenerate.

Somatic cells can be reprogrammed to a pluripotent state by overexpressing the four Yamanaka factors (Oct-3/4, Sox2, KLF4, and c-Myc, hereafter referred to as 4F; Takahashi et al., 2007; Takahashi and Yamanaka, 2006) for several weeks. Although systemic 4F overexpression can induce dedifferentiation even *in vivo*, the end result in most instances is cancer (Abad et al., 2013; Ohnishi et al., 2014; Shibata et al., 2018). We therefore switched to a short-term 4F induction protocol and demonstrated that 4F ameliorates aging processes in a mouse model of premature aging (Ocampo et al., 2016). Recent studies showed that *in vivo* reprogramming can improve regeneration of muscle, optic nerve, and cardiomyocytes (Chen et al., 2021; Lu et al., 2020; Wang et al., 2021). However, it remains an open question whether short-term 4F induction can transiently and partially reprogram mature cells to a plastic state *in vivo* to promote the regeneration of mammalian tissues without also driving tumor formation. This question is difficult to answer without a stringent lineage-tracing system but is important to address since an increase in regenerative capacity could, in principle, be harnessed to treat many human diseases.

With this in mind, here, we developed a mouse model that enables both the inducible expression of 4F in specific tissues, as well as the ability to track 4F-expressing cells. Although tissue regeneration in mammals generally is poor, the liver, if not severely injured, has some regenerative abilities. Thus, we decided to focus our question on the liver and therefore used hepatocyte-specific 4F (Hep-4F) mice for all subsequent experiments.

## RESULTS

### Hepatocyte-specific 4F expression induces cell proliferation and the loss of hepatic characteristics

**Figure 1.**
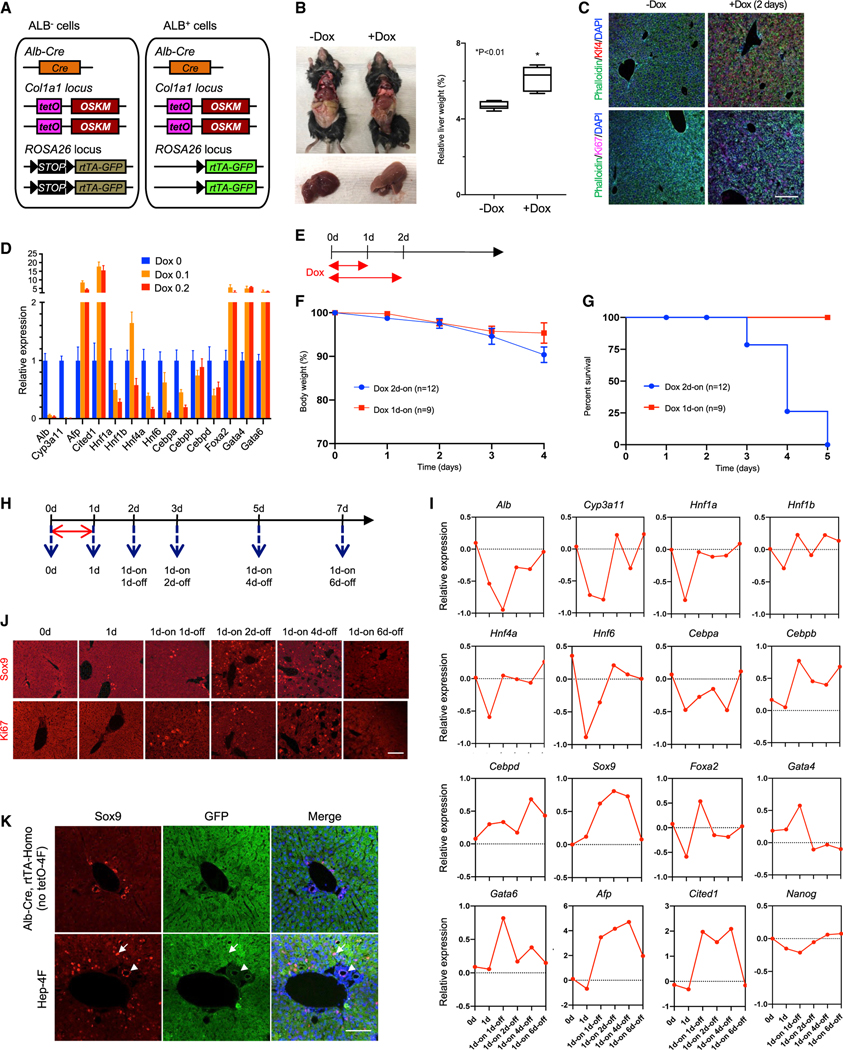
Induction of transient, partial reprogramming by liver-specific 4F expression (A) Schematic representation of the genetic makeup for lineage-traceable, liver-specific 4F inducible mouse models. In this model, rtTA can be activated by Alb-Cre, allowing for specific 4F induction in the liver in a Tet-ON manner. (B) Livers collected from Dox-treated and untreated Hep-4F mice 2 days after Dox administration. Left: representative images are shown. Right: relative liver weight (% body weight) is shown. Data represent the mean with SE (n = 5). *p < 0.01 (unpaired t test). (C) Immunostaining for Klf4 and Ki67. Livers were collected 2 days after Dox administration. Scale bar, 200 μm. (D) qPCR analysis for liver-related genes in the liver of Hep-4F mice treated with different concentrations of Dox (0.1 or 0.2 mg/mL) for 2 days. Data represent the mean with SD (n = 3; technical replicates). (E) Schematic representation for Dox treatment protocol. (F and G) Body weight (F) and survival (G) of Hep-4F mice after Dox treatment (0.1 mg/mL; 1d-on, n = 9; 2d-on, n = 12). (H) Schematic representation of time course for Dox treatment protocol. (I and J) Time course experiment of qPCR (I) and IHC for Sox9 and Ki67 (J). Data represent the mean (n = 2; biological replicates). Scale bar, 200 μm. (K) GFP-based lineage-tracing experiments for hepatocytes after 4F induction. Livers were collected 1 day after Dox withdrawal. White arrow and white arrowhead indicate atypical Sox9^+^ cells and Sox9^+^ cholangiocytes, respectively. Scale bar, 100 μm.

The Hep-4F mouse model includes the albumin (Alb)-Cre transgene, allowing for liver-specific Cre recombinase expression, tetO-4F, and LoxP-STOP-LoxP-rtTA-IRES-GFP (Figure 1A). In this mouse model, the LoxP-STOP-LoxP cassette is excised by Alb-Cre, resulting in the expression of rtTA and GFP in hepatocytes, which enables lineage tracing. Thus, administration of doxycycline (Dox) allows for liver-specific 4F expression. We first treated Hep-4F mice with Dox for 2 days. Quantitative RT-PCR (qRT-PCR) analysis of collected tissues confirmed that 4F induction was specific to the liver (Figure S1A). We also observed that 4F expression resulted in a large, pale liver (Figure 1B). Any noticeable histological change was not observed in the lung and kidney, in which negligible gene expression of 4F was observed after Dox treatment (Figure S1B). Immunostaining for Ki67, a proliferation marker, showed that the number of Ki67-positive proliferating cells was increased 2 days after Dox treatment (Figure 1C). Thus, 4F quickly promoted the proliferation of liver cells. We next examined the expression of liver-specific marker genes via qRT-PCR (Figure 1D). Markers of mature hepatocytes (*Alb* and *Cyp3a11*) and differentiated liver-enriched transcription factors (*Hnf1a, Hnf4a,* and *Hnf6*) were downregulated, whereas *Foxa2*, *Gata4*, and *Gata6*, which play more important roles during liver ontogeny, were upregulated. Interestingly, the gene *Afp*, which encodes a serum protein that is normally silenced after birth, was also upregulated, both at the transcription and protein levels (Figures 1D and S1C). This may also indicate cellular proliferation, as Afp is elevated during liver regeneration (Nakano et al., 2017).

Two days of Dox treatment caused the mice to die within 5 days. A failure of liver function was identified as one possible reason for mouse death, because mature hepatocyte markers, such as Alb and Cyp3a11, were downregulated. To investigate that possibility, comprehensive metabolic panel analyses were performed (Figure S1D). These metabolic analyses indeed showed poor liver function, and therefore, liver failure caused by 4F may be one reason for mouse death.

To optimize the experimental condition where lethal effects can be minimized, we systematically varied the amount of Dox that was administered, as well as the duration of treatment. Limiting Dox treatment to 1 day at 0.1 mg/mL allowed the Hep-4F mice to survive (Figures 1E–1G). We then used this protocol to perform time course experiments, collecting liver samples at the indicated time points (Figure 1H). Dox treatment downregulated differentiated hepatocyte markers and upregulated *Gata4*, *Gata6*, *Foxa2*, and *Sox9* (Figure 1I). The dedifferentiation effect of 4F appeared to be transient, as the expression of adult hepatocyte markers returned to normal levels after Dox withdrawal. In contrast to previous studies where the dose and timing was higher (Abad et al., 2013; Ohnishi et al., 2014; Shibata et al., 2018), this transient liver-specific 4F induction never resulted in tumor formation (data not shown). This analysis was performed up to 9 months after Dox withdrawal, although any tumorigenic activity mediated by 4F will need to be further investigated. Similarly, the effect of short-term 4F on cellular proliferation was also relatively transient (Figure 1J), showing a correlation between the loss of differentiated hepatocyte markers and proliferation. To see whether hepatic zonation is altered by 4F induction, immunostaining was performed for E-cadherin (E-Cad) and glutamine synthetase (GS) as zonally expressed markers for zone 1 and zone 3, respectively (He et al., 2021; Wei et al., 2021; Figure S1E). It seems that the impact of 4F on zonation was minimal. We also found that Sox9^+^ cells diffusely emerged in the liver after Dox treatment in a transient manner (Figures 1J and S1F). Importantly, these Sox9^+^ cells were GFP positive and distinct from GFP^dim^ cholangiocytes, which are epithelial cells of the bile duct that also express Sox9 (Athwal et al., 2017; Figure 1K). This suggests that 4F expression partially reprogrammed hepatocytes to Sox9^+^ cells. Levels of Sox9 expression remained elevated following Dox withdrawal, suggesting that 4F may induce dedifferentiation to an undetermined plastic state and that these cells are then redirected back to the hepatocyte lineage (following Dox withdrawal) by the surrounding niche. This will require further investigation.

We next tested whether Myc alone, which is known to induce hepatocyte proliferation (Shachaf et al., 2004), is sufficient to induce loss of mature hepatocyte markers. For this analysis, we used a Hep-Myc mouse model carrying Alb-Cre, tetO-MYC (human), and LSL-rtTA-IRES-GFP. qRT-PCR results showed that, unlike 4F, MYC did not induce the loss of mature hepatocyte markers (Figure S1G), suggesting that partial reprogramming does not solely depend on cell proliferation.

### Global analysis of chromatin accessibility and gene transcription revealed that 4F induces partial reprogramming *in vivo*

**Figure 2.**
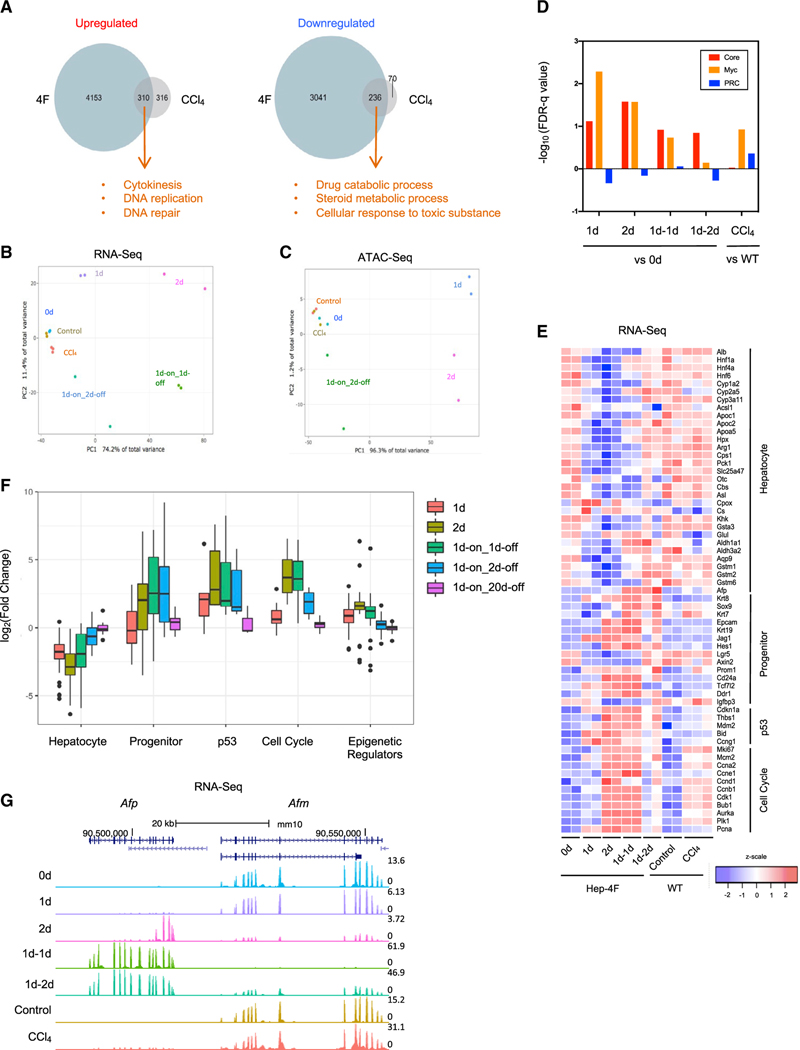
Global analysis of transcriptome and chromatin accessibility of Hep-4F mice (A) Venn diagrams summarizing overlapping upregulated or downregulated genes responsive to 4F expression (day 2 versus day 0) and CCl_4_ treatment. GO analyses were performed for overlapping DEGs. (B and C) PCA analysis for RNA-seq (B) and ATAC-seq (C). Livers were collected at the indicated time points: 0d, 1d, 2d, 1d-on_1d-off, and 1d-on_2d-off. To compare reprogramming with regeneration, B6 mice were treated with or without CCl4 for acute injury, and the livers were collected 3 days after the treatment: control and CCl4. Each sample was prepared in duplicates except CCl4 samples, which are in triplicates. (D) Bar chart for false discovery rate (FDR)-q value for ESC modules (core, Myc, and PRC modules) as calculated from RNA-seq. WT, wild type. (E) Heatmap for liver gene-expression signature in Hep-4F mice. (F) Long-term effect of 4F after Dox withdrawal. Boxplots show gene expression of each gene set (related to Figures 2E and 3B), normalized to that of control samples (0 days) based on RNA-seq. (G) Genome browser tracks of RNA-seq for *Afp* and *Afm* loci.

We next performed global transcriptomic and DNA accessibility analyses using RNA sequencing (RNA-seq) and an assay for transposase-accessible chromatin using sequencing (ATAC-seq). As a reference, we included liver samples collected from mice injected with carbon tetrachloride (CCl_4_), which causes acute liver injury followed by liver regeneration (Bezerra et al., 1999; Nakano et al., 2017). Consistent with previous reports, CCl_4_ administration activated genes related to cell proliferation. These genes partially overlapped with the genes activated by 4F expression (Figure 2A). We performed principal-component analysis (PCA) of the RNA-seq and ATAC-seq data (Figures 2B and 2C). CCl_4_ treatment clearly affected gene expression but had little if any effect on chromatin accessibility. In sharp contrast, 4F dramatically changed chromatin accessibility, presumably leading to massive alterations in gene expression. We next performed unbiased clustering of ATAC-seq peaks, resulting in six clusters (Figure S2A). Two of these six clusters were associated with a downregulation of DNA accessibility after Dox treatment, suggesting that these regions switched from an “open to closed” chromatin state (OC). The other clusters switched from “closed to open” (CO), with variation in the timing of this switch. We performed motif analysis, revealing that motifs associated with hepatocyte-enriched transcription factors were largely enriched in the OC1 cluster and that 4F motifs were enriched in CO1/2 groups (Figure S2B). Of note, motifs for Jun/Fos (AP1 complex), Gata4/6, and Foxa2 were generally activated by Dox treatment. These pioneer factors (Biddie et al., 2011; Iwafuchi-Doi et al., 2016; Zaret et al., 2016) may have contributed to the 4F-mediated global changes in chromatin accessibility. Taken together, these data indicate that 4F induces reprogramming, global changes in DNA accessibility, and subsequent changes to the hepatocyte transcriptional signature.

We next analyzed RNA-seq data in detail. A previous report showed that the embryonic stem cell (ESC) transcription signature can be dissected into three modules: core, Myc, and PRC modules (Kim et al., 2010). Focusing on these modules, we found that core and Myc modules were transiently activated, indicating that 4F was able to activate the ESC program in liver cells (Figure 2D). However, core pluripotency factors, such as Nanog, *Utf1*, and *Rex1*, were not expressed (data not shown). Of note, CCl_4_ did not induce core modules but was able to affect Myc modules to some degree, consistent with previous reports that *c*-*Myc* is upregulated during liver regeneration (White et al., 2005). We next assessed hepatocyte-related genes. Markers of mature hepatocytes were globally downregulated soon after Dox administration (Figures 2E and S3). At the same time, cell-cycle-related genes were being activated (Figure 2E). Expression of liver progenitor and stem cell markers, such as Sox9 and Epcam, were upregulated (Figure 2E) together with increases in chromatin accessibility within putative enhancer regions (Figure S2C). Of note, such changes induced by 4F were almost reverted 20 days after Dox withdrawal as analyzed by RNA-seq (Figure 2F). This is consistent with 4F inducing partial reprogramming in the liver in a transient manner.

Alb, Afp, and Afm are tandemly arranged in the same transcriptional orientation, although little is known about how these genes are regulated (Jin et al., 2009). Interestingly, our RNA-seq data revealed that Afp and Afm are reciprocally regulated (Figure 2G). Since Afp expression is correlated with cancers (Chen et al., 2020; Mizejewski, 2002), we asked whether this reciprocal regulation is observed in hepatocellular carcinoma (HCC) using public data. We analyzed 313 cases and found that there is a reverse correlation between AFP and AFM (p = 0.036; Figure S4A). More importantly, the AFP^low^AFM^high^ group had a higher survival rate than the AFP^high^AFM^low^ group (p = 0.011; Figure S4B), indicating the importance of the reciprocal regulation between AFP and AFM in the context of cancer.

**Figure 3.**
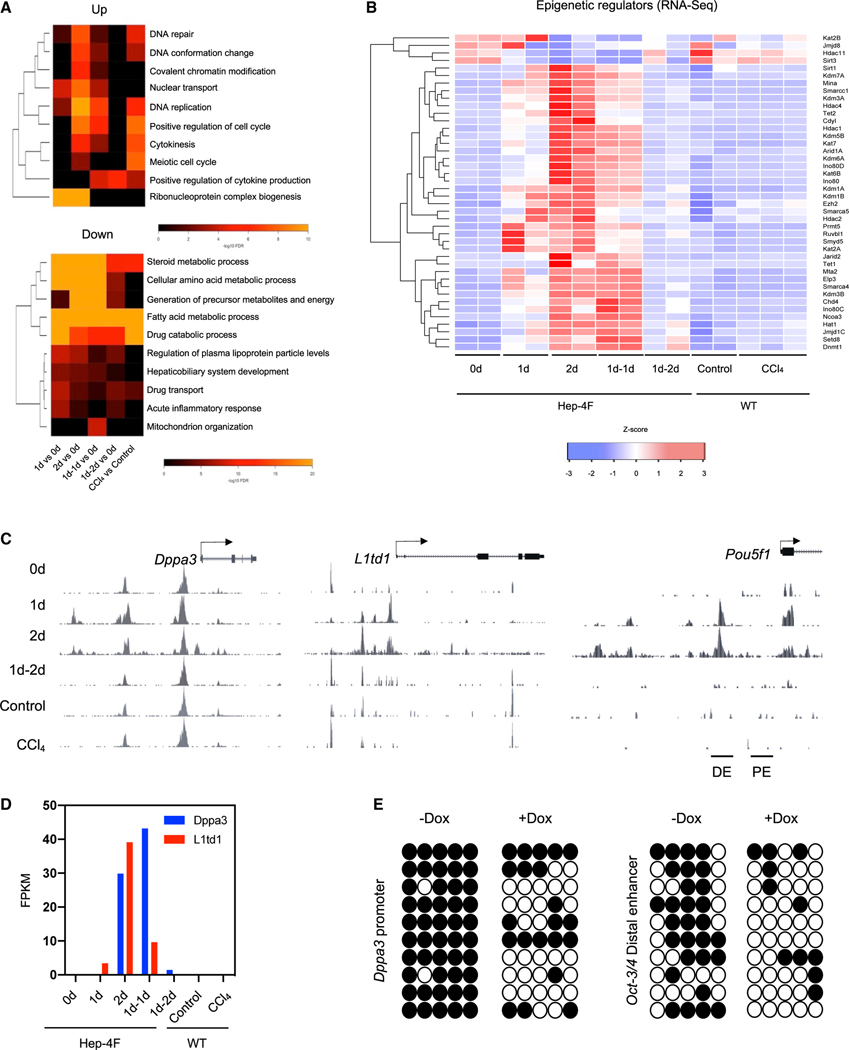
Epigenetic reprogramming by liver-specific 4F expression (A) GO analysis for differentiation-expressed genes in RNA-seq. (B) Heatmap for epigenetic modifiers in Hep-4F mice. (C) Genome browser view of the ATAC-seq data at the indicated gene loci. (D) Expression levels of *Dppa3* and *L1td1* genes in Dox-treated Hep-4F mice. Data were extracted from RNA-seq (Figure 2). Data represent the mean (n = 2; biological replicates). FPKM, fragments per kilobase of exon per million mapped fragments. (E) Bisulfite sequencing of the *Dppa3* promoter and distal enhancer of *Oct-3/4* with the liver samples collected from the mice treated with or without Dox for 2 days.

We next performed Gene Ontology (GO) analysis for differentially expressed genes (DEGs) (Figure 3A). Cell-cycle-related GO terms, such as DNA replication and cytokinesis, were upregulated, whereas metabolic pathway GO terms were downregulated. Interestingly, epigenetic-modification-related terms, such as DNA conformation change and covalent chromatin modification, were also upregulated, driving us to investigate epigenetic modifiers. Indeed, 4F induced lots of epigenetic modifiers (Figure 3B), which would explain 4F-mediated global changes in DNA accessibility. With the RNA- and ATAC-seq data, we found that 4F activated several silenced genes, such as *Dppa3* and *L1td1*, as well as the distal enhancer of *Oct-3/4* (Figures 3C and 3D). During induced pluripotent stem cell (iPSC) reprogramming *in vitro*, these genes are not activated until the pluripotent state is established (Xu et al., 2015), indicating that *in vivo* reprogramming is unique (e.g., *in vivo* reprogramming induces totipotency; Abad et al., 2013). Consistent with the activation of these genes, DNA methylation analysis with bisulfite conversion revealed that Dox treatment reduced levels of DNA methylation within the *Dppa3* and *Oct-3/4* loci (Figure 3E). Taken together, 4F transiently induced very notable changes in chromatin, supporting the notion that 4F mediates epigenetic reprogramming.

**Figure 4.**
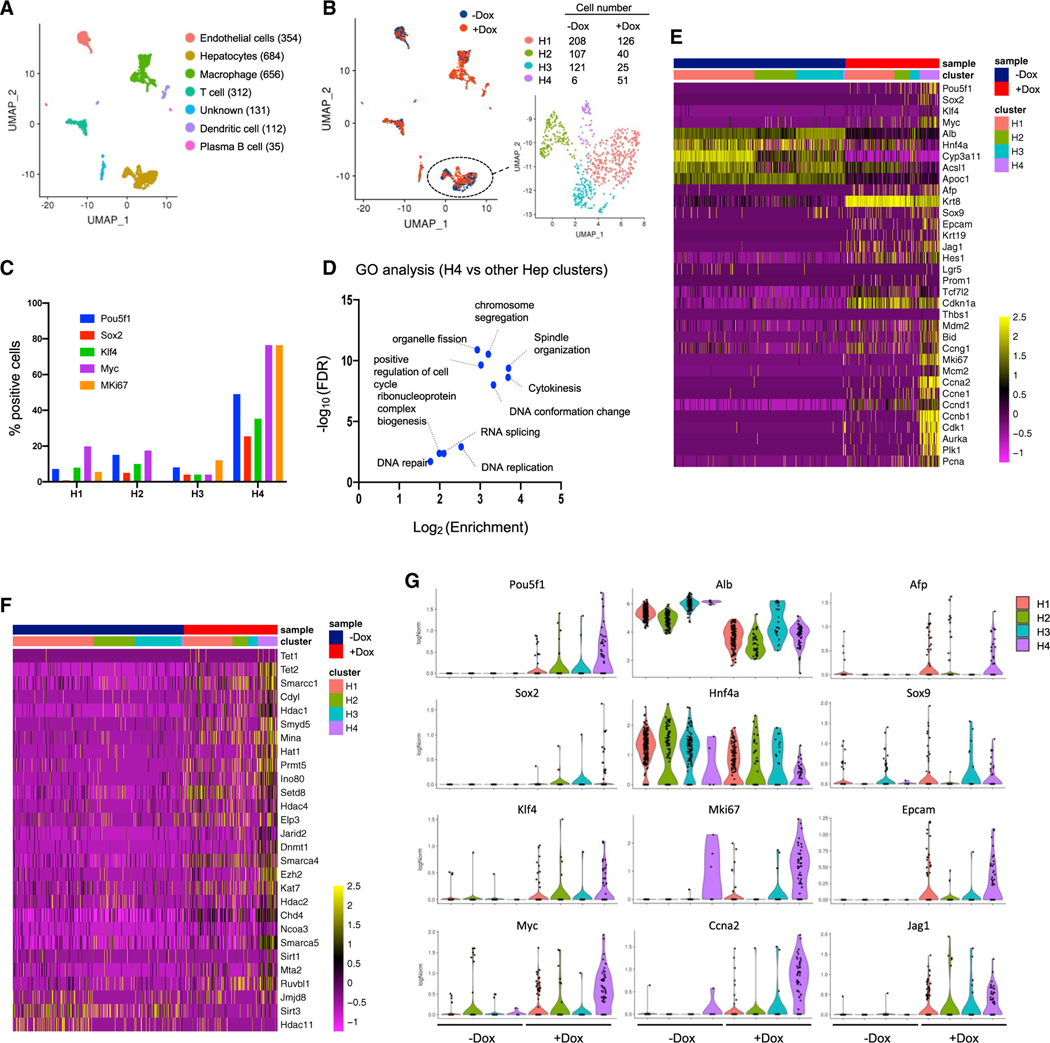
Single-cell transcriptome analysis of Hep-4F mice (A) UMAP visualization of liver cell clusters. Hep-4F mice were treated with or without Dox for 1 day and single-cell suspensions were prepared from the liversisolated at 1 day after Dox withdrawal. Each cell type was characterized based on gene expression (Figure S5A). (B) Left: UMAP plot of untreated (−Dox) or Dox-treated (+Dox) liver cell clusters: −Dox (blue dots) and +Dox (red dots). Right: enlarged hepatocyte clusters are shown. (C) Relative cell number for the indicated genes in each hepatocyte cluster. (D) Volcano plots of GO analyses for DEGs between H4 cells and the other hepatocyte cells. (E and F) Heatmap of gene expression of the liver-related and cell-cycle-related genes (E) and of the epigenetic modifiers (F) in the cells of hepatocyte subclusters. (G) Violin plots for gene expression of the reprogramming-related genes in each hepatic cluster.

We next performed single-cell RNA-seq (scRNA-seq), collecting liver samples 1 day after Dox withdrawal (1d-on_1d-off). We obtained 2,284 single-cell transcriptomes (−Dox: 1,258 cells; +Dox: 1,026 cells). Uniform manifold approximation and projection (UMAP) plots revealed seven clusters, which could be identified based on marker gene expression (Figures 4A and S5A). Dox treatment induced 4F expression specifically in hepatocyte populations and changed their gene expression signatures (Figures 4B and S5B). The gene *Alb* was downregulated, inversely correlating with 4F expression (Figure S5C). DEGs for each cell type were next analyzed (Figure S5D). These analyses show that hepatocytes have the highest number of DE genes, indicating that these cell types are the most affected by 4F induction. Upregulated genes in Dox-treated condition are specific to hepatocytes, while some common signatures (liver metabolism related) were observed in downregulated genes across cell types. Macrophages and endothelial cells were most affected among non-hepatocyte cell types in transcriptomes. Consistent with these data, Dox-treated livers had fewer endothelial cells and more macrophages, which may be a secondary effect of hepatocyte proliferation. The hepatocyte population could be further subdivided into four clusters (H1, H2, H3, and H4; Figure 4B). H4 cells were more prevalent in the Dox-treated condition (Figure 4B) and expressed high levels of 4F, proliferation-related genes, and epigenetic modifiers, suggesting that the H4 cluster exhibits the strongest signature of reprogramming (Figures 4C–4F). We also found that Dox-treated hepatocytes upregulated p53 target genes, such as *Cdkn1a* and *Mdm2*. This is consistent with a previously described compensatory mechanism, as 4F has been shown to activate the p53 pathway (Hong et al., 2009; Kawamura et al., 2009). Finally, Dox-treated H3 cells exhibited partial restoration of gene signatures associated with mature hepatocytes (Alb and Cyp3a11) and had lower levels of *cdkn1a* expression (Figures 4E and 4G). Thus, short-term 4F may induce partial reprogramming and cellular proliferation, but after Dox withdrawal, the cells begin to restore normal hepatic characteristics, such as quiescence. Our results hint at a possible cell trajectory after partial reprogramming in the liver, although we cannot distinguish between cells that are on their way back to normal versus those that were never strongly reprogrammed to begin with.

### Top2a is required for reprogramming *in vitro* and *in vivo*

**Figure 5.**
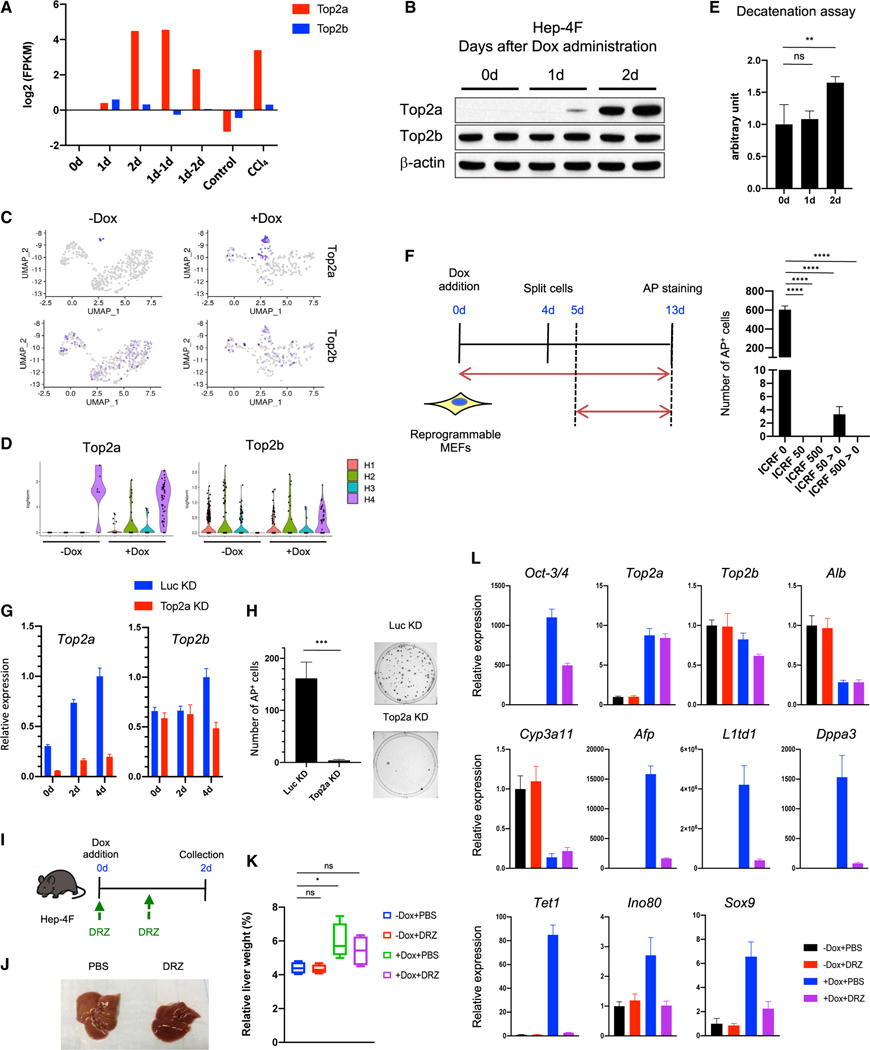
Role of Top2a on reprogramming *in vitro* and *in vivo* (A) Time course for gene expression of Top2a and Top2b in reprogramming livers. Data were extracted from RNA-seq (Figure 2). Data represent the mean (n = 2; biological replicates). (B) Western blotting for Top2a and Top2b in liver samples collected at indicated time points after Dox administration. (C) UMAP visualization for Top2a and Top2b. (D) Violin plots for gene expression of Top2a and Top2b in each hepatic cluster. (E) Decatenation assay for topoisomerase activity in liver samples. Data represent the mean with SD (n = 4). ns, not significant. (F) Effect of ICRF-193 (Top2 inhibitor) on iPSC reprogramming. Left: schematic representation for ICRF-193 (ICRF) treatment protocol is shown. Right: reprogramming efficiency is shown. Data represent the mean with SD (n = 3). (G) qPCR for Top2a and Top2b in the cells expressing short hairpin RNA (shRNA) for Top2a (Top2a knockdown [KD]) or for Luciferase (Luc KD). Data represent the mean with SD (n = 3; technical replicates). (H) Reprogramming efficiency of Top2a or Luc KD cells. Data represent the mean with SD (n = 3). (I) Schematic representation of dexrazoxane (DRZ) treatment protocol. (J) Livers collected from PBS-treated and DRZ-treated Hep-4F mice 1 day after Dox withdrawal. (K) Relative liver weight (% body weight). Data represent the mean with SE (n = 5). (L) qPCR analysis for PBS- or DRZ-treated Hep-4F mice with or without Dox for 2 days. Data represent the mean with SD (n = 3; technical replicates). Statistical analyses were conducted by unpaired t test or one-way ANOVA with Tukey’s post hoc analysis. *p < 0.05; **p < 0.01; ***p < 0.001; ****p < 0.0001.

We found that *in vivo* reprogramming rapidly induces the expression of epigenetic genes. We therefore hypothesized that epigenetic regulators might be involved in partial reprogramming (Figures 3B and 4F). Since the mechanisms of partial reprogramming are largely unknown and this is an important knowledge gap that must be addressed before therapeutic application, we investigated these downstream factors in more detail. Among them, topoisomerase2a (Top2a) was a good candidate for mediating 4F’s function because its expression is largely restricted to development, playing crucial roles in regulating the epigenome (Miller et al., 2017; Thakurela et al., 2013). Moreover, Top2a, not Top2b, was highly induced by 4F at mRNA and protein levels and scRNA-seq showed Top2a induction was more specific to the reprogrammed population (H3 cluster; Figures 5A–5D). Moreover, Top2 activity increased at day 2 after 4F induction as assessed by decatenation assay (Figure 5E), which prompted us to investigate the role of Top2a on cellular reprogramming. To do so, we first treated reprogrammable mouse embryonic fibroblasts (MEFs) with Dox in the presence or absence of ICRF-193, a Top2 inhibitor, which inhibits both TOP2A and TOP2B (Tanabe et al., 1991). ICRF-193 treatment blocked iPSC reprogramming in all conditions tested (Figure 5F). Since Top2a expression is induced by 4F (unlike Top2b), we performed RNAi experiment for Top2a (Figure 5G). RNAi-mediated knockdown led to a 40-fold reduction in reprogramming efficiency, highlighting the crucial role of Top2a in reprogramming (Figure 5H). We next asked whether Top2 inhibition affects *in vivo* reprogramming. To do so, we treated Hep-4F mice with Dox in the presence of PBS or dexrazoxane hydrochloride (DRZ), another Top2 inhibitor, which inhibits both isoforms TOP2A and TOP2B. We used DRZ because it can be dissolved in PBS and therefore is more suitable for *in vivo* purposes (Hasinoff et al., 1997; Figure 5I). DRZ inhibited the change in the color of the liver (Figure 5J), although it did not affect relative liver weight (Figure 5K). DRZ blocked the induction of progenitor markers and epigenetic regulators, while the loss of mature hepatic markers was not affected (Figure 5L). DRZ treatment itself did not affect gene expression (data not shown). Collectively, our data show that *in vitro* and *in vivo* reprogramming depends on Top2a.

### *In vivo* reprogramming has beneficial effects on regenerative capacity

**Figure 6.**
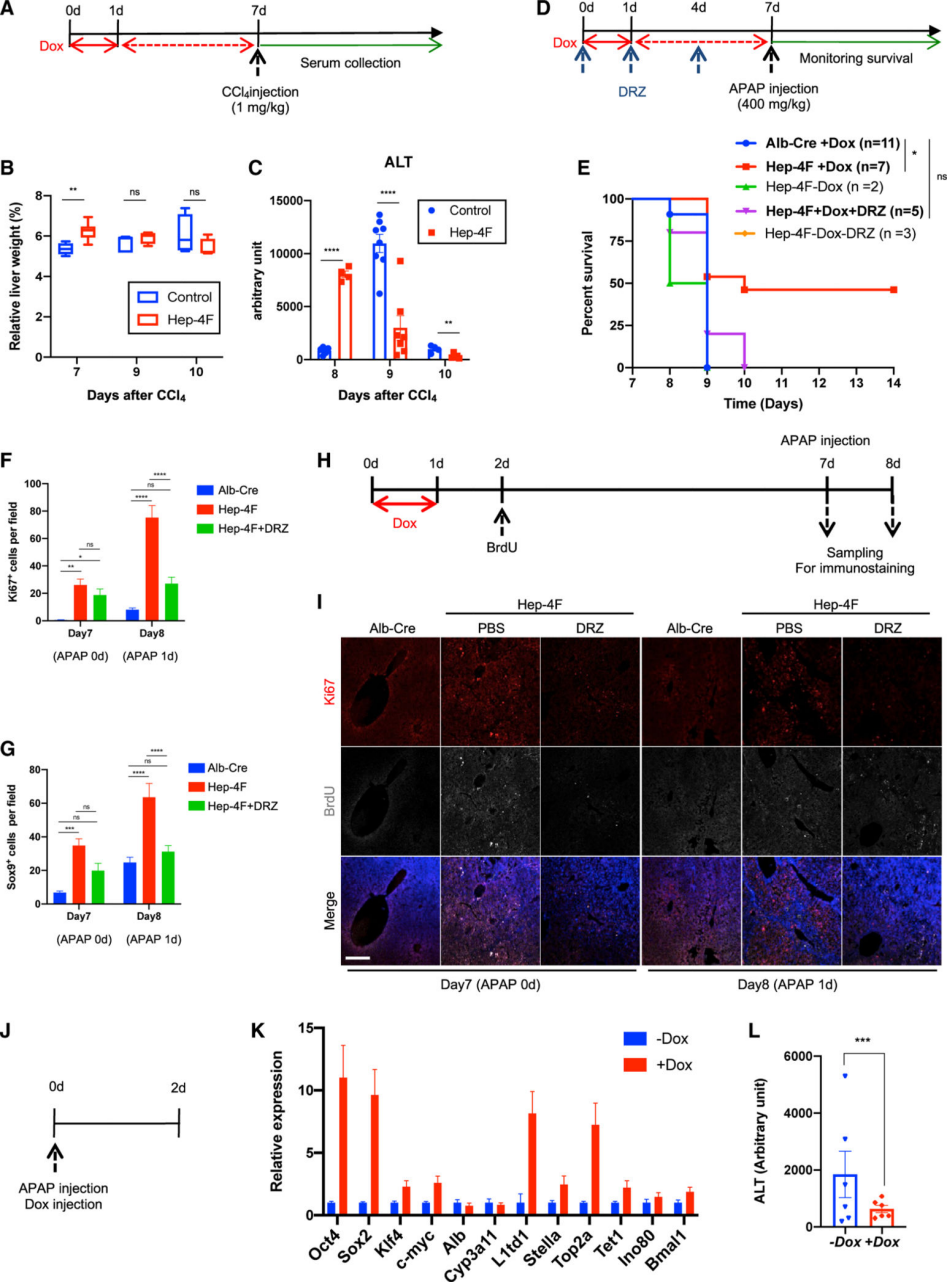
Improvement of liver regeneration capacity induced by 4F-mediated partial reprogramming (A) Schematic representation for CCl_4_ treatment protocol. Alb-Cre mice (no 4F cassette) or Hep-4F mice were treated with Dox for 1 day. Six days following Dox withdrawal, the mice were injected with CCl_4_. (B) Relative liver weight (% body weight). Data represent the mean with SE (n = 5). (C) ALT levels of Alb-Cre or Hep-4F mice. Data represent the mean with SE (control: n = 5 [8 days], n = 8 [9 days], n = 4 [10 days]; Hep-4F, n = 4 [8 days], n = 7 [9 days], n = 5 [10 days]). (D) Schematic representation for APAP treatment protocol. Hep-4F mice were treated with or without Dox for 1 day. Six days following Dox withdrawal, the mice were injected with APAP. (E) Survival curve of Hep-4F mice. (F) Quantification of Ki67^+^ cells. Data represent the mean with SE (n = 12). (G) Quantification of Sox9^+^ cells. Data represent the mean with SE (n = 12). (H) Schematic representation for APAP treatment protocol for cell tracking of BrdU-positive cells. (I) IHC for Ki67 and BrdU. Scale bar, 200 μm. (J) Schematic representation for the protocol of simultaneous injection of Dox and APAP. (K) qPCR analysis for Hep-4F mice with or without Dox for 2 days. Data represent the mean with SD (n = 3; technical replicates). (L) ALT levels of Dox-treated or untreated Hep-4F mice 2 days after APAP treatment. Data represent the mean with SE (n = 6). Statistical analyses were conducted by unpaired t test or one-way ANOVA with Tukey’s post hoc analysis.*p < 0.05; **p < 0.01; ***p < 0.001; ****p < 0.0001.

We next asked whether 4F induction could have beneficial effects on the liver. To do so, we treated 1-day-Dox-treated Hep4F mice with CCl4 to monitor alanine transaminase (ALT), a marker of liver injury (Figure 6A). We observed an earlier clearance of ALT in Dox-treated Hep-4F mice and an earlier increase in relative liver weight (Figures 6B and 6C). We next administered a lethal dose of acetaminophen (APAP) to Hep-4F mice. APAP is commonly used to study acute liver injury (Jaeschke et al., 2014). In this model, liver regeneration is tightly associated with survival, as mice die rapidly without sufficient liver regeneration (Bhushan and Apte, 2019). Hep-4F mice were treated with Dox for 1 day and then APAP was administered 6 days later (day 7) following Dox withdrawal (Figure 6D). Two days after APAP administration, all control mice died, but half of the Dox-treated mice survived (Figure 6E). Associated with longer survival of Dox-treated Hep-4F mice (Figure 6E), Dox-treated livers were more proliferative compared with control livers before and after APAP treatment (Figure 6F). Importantly, this beneficial effect of 4F was not observed when mice were treated with DRZ (Figure 6E), highlighting the importance of Top2a for liver regeneration. DRZ treatment did not affect cell proliferation before APAP treatment; however, it resulted in a 2-fold reduction in Ki67^+^ cells along with the decrease in Sox9^+^ cells (Figures 6F and 6G). Top2a may be involved in 4F enhancing regeneration competency in a cell-proliferation-independent mechanism, which might be attributed to epigenetic reprogramming. Further analyses are needed to clarify this mechanism.

We next asked whether the partially reprogrammed, proliferative hepatocytes that express 4F can contribute to liver regeneration. To measure this, we added a bromodeoxyuridine (BrdU) injection on day 2 of our APAP protocol and assessed whether BrdU^+^ cells expressed Alb (Figure S6A). Alb and BrdU signals overlapped, suggesting that BrdU-labeled cells contributed to liver regeneration (Figure S6B). Ki67^+^ cells were largely labeled with BrdU before APAP treatment; however, Ki67^+^ cells that emerged post-APAP-mediated injury were not always positive for BrdU (Figures 6H and 6I), suggesting that enhancing effect of 4F on proliferation may not simply explain the increase in cell number prior to liver injury and may be mediated by other mechanisms, where, for instance, the cells that did not express 4F but proliferate in a compensatory mechanism might contribute to the regeneration. RNA-seq was performed for the liver samples collected before and after APAP treatment. DEG analyses show that GO terms associated with cell cycle and proliferation were enriched in Hep-4F mice consistent with better regeneration (Figure S6C), although this may not be the cause but rather a consequence of better regeneration.

Lastly, to test whether 4F can have a beneficial effect even when 4F is induced on or after liver injury, we treated mice with APAP and Dox simultaneously (Figure 6J). The expression of 4F and epigenetic regulators, such as Top2a, was still induced in this context (Figure 6K). Importantly, Dox-treated mice showed lower levels of ALT (Figure 6L). Taken together, *in vivo* reprogramming shows beneficial effects by promoting the regeneration of injured liver. Our study is therefore an important step toward developing partial reprogramming therapies for treating human diseases.

## DISCUSSION

In summary, here, we have developed a mouse model that enables hepatocyte-specific 4F induction and subsequent lineage tracing of 4F-expressing cells. We demonstrate that liver-specific 4F expression rapidly and transiently induced partial reprogramming and that this enhanced liver regeneration. This study, the first to perform lineage tracing and single-cell transcriptome analyses for 4F-expressing cells *in vivo*, shows that 4F-mediated cellular partial reprogramming is a potential avenue for inducing a proliferative, plastic progenitor state.

It was originally reported that systemic 4F expression *in vivo* leads to cancers with totipotency (Abad et al., 2013). Subsequent reports demonstrated the beneficial effects of 4F in several different contexts (Doeser et al., 2018; Lu et al., 2020; Ocampo et al., 2016; Browder and Reddy, 2022). Our previous report showed that cyclic 4F expression ameliorates age-associated hallmarks in a mouse model of premature aging (Ocampo et al., 2016). Another recent paper showed that expression of Oct-3/4, Sox2, and KLF4 in the retina restores youthful DNA methylation patterns and transcriptomes and promotes axon regeneration after injury (Lu et al., 2020). However, it remains unknown whether short-term 4F induction leads to dedifferentiation *in vivo* because of a lack of a stringent system for lineage tracing. Here, we demonstrate that 4F-expressing cells partially reprogram to progenitor state, associated with cell proliferation in the liver consistent with the report that dedifferentiation is associated with cell proliferation.

Our findings raise several questions, namely whether the effect of 4F is tissue or context specific and what is the molecular basis of quick reprogramming induced by 4F? Further study will be needed to thoroughly answer these questions, but here, we report that Top2a is a critical component of the mechanism underlying *in vivo* reprogramming. Inhibition of Top2 activity dramatically reduced the beneficial effect of 4F and inhibited the increase in gene expression of epigenetic regulators and progenitor markers while not affecting the loss of mature hepatocyte markers. This suggests that Top2a-mediated partial cellular reprogramming is required for 4F-mediated benefits. Notably, Top2a inhibition dramatically suppressed the expression of Tet1, which was recently reported to have a crucial role in reprogramming-mediated regeneration induced by 4F (Lu et al., 2020), implying that Top2a may act upstream of epigenetic modifiers, such as Tet1.

We observed a strong correlation between the loss of mature hepatic markers and proliferation in reprogramming hepatocytes. This highlights a causal link between dedifferentiation and proliferation, which is consistent with previous reports where dedifferentiation followed by proliferation contributes to tissue regeneration in vertebrate species with high regenerative capacities (Jopling et al., 2010; Tanaka et al., 2016). Therefore, *in vivo* 4F-mediated partial reprogramming may share some of the mechanisms that underlie the tissue and organ regeneration observed in nature. Further analyses of dedifferentiation mechanisms should lead to new strategies for expanding the regenerative capacity of mammalian tissues.

### Limitations of the study

While we demonstrated that hepatocyte-specific 4F induction enhances liver regenerative capacity by partial reprogramming mediated through topoisomerase2, this study still has several limitations. First, prevalent chronic liver injuries, such as fibrosis and non-alcoholic fatty liver disease, were not tested here but should be addressed in future projects. Second, longevity and tumorigenic risks were not fully assessed with longer term monitoring of Dox-treated Hep-4F mice, and evaluating maximum lifespan and tumor incidence after Dox treatment will be required for complete safety assessment. Third, mechanistically, it remains to be elucidated how Top2a can participate in hepatocyte cellular reprogramming and whether Top2a activation alone is sufficient to enhance liver regeneration. Lastly, cell trajectories of reprogramming hepatocytes were not fully investigated, and for now, it remains unclear how these reprogrammed cells contribute to the enhancement of liver regeneration in detail. Deeper single-cell analyses with additional *in vivo* tracing systems for the partially reprogrammed cells will help understand their behaviors during liver regeneration.

## STAR★METHODS

### RESOURCE AVAILABILITY

#### Lead contact

Further information and requests for resources and reagents should be directed to and will be fulfilled by the lead contact, Juan Carlos Izpisua Belmonte (belmonte@salk.edu).

#### Materials availability

All unique/stable reagents/cells generated in this study are available from the lead contact with a completed Materials Transfer Agreement.

#### Data and code availability

- RNA-Seq, ATAC-Seq and sc-RNAseq data have been deposited to Gene Expression Omnibus and are available to the public as of data of publication. The accession number is listed in the key resources table.
- This paper does not report any original code.
- Any additional information required to reanalyze the data reported in this paper is available from the lead contact upon request.

### EXPERIMENTAL MODEL AND SUBJECT DETAILS

#### Mice

*Alb-Cre* (Postic et al., 1999), *Col1a1*^tetO–4F/tetO–4F^;*ROSA26*^rtTA/rtTA^, *Col1a1*^2loxP-tetO–4F/2loxP-tetO–4F^;*ROSA26*^rtTA/rtTA^ (Carey et al., 2010), tetO-MYC (Felsher and Bishop, 1999), and *ROSA*^LSL-rtTA-IRES-GFP/LSL-rtTA-IRES-GFP^ (Belteki et al., 2005) have been previously described. These mice were purchased from the Jackson laboratory. The mice were housed in a 12 h light/dark cycle (light between 06:00 and 18:00) in a temperature-controlled room (22 ± 1C) with free access to water and food. All procedures were performed in accordance with protocols approved by the IACUC and Animal Resources Department of the Salk Institute for Biological Studies. Two-to four-months old mice were experimentally used. Both female and male mice were used and no notable sex-dependent differences were found in our analyses.

Dox was administered in drinking water (0.1 mg/mL) unless otherwise stated. For acute liver injury, the mice were intraperitoneally injected with CCl_4_ (Sigma) (5 ul/g mouse of 10% CCl_4_ in corn oil) once and the livers were collected 3 days after the injection. For liver injury, APAP (350 mg/kg) was intraperitoneally injected to the mice. All animal experiments were approved by the IACUC committee and conform to regulatory standards.

#### Cell lines and cell culture

MEFs were prepared from reprogrammable mice (*Col1a1*^2loxP-tetO–4F/2loxP-tetO–4F^;*ROSA26*^rtTA/rtTA^) with the standard protocol. Briefly, E13.5 embryos were isolated, and their heads and visceral tissues were removed. After washing the remaining bodies with PBS, the bodies were minced with scissors in 0.25% trypsin-EDTA and incubated at 37°C for 20 min. DMEM containing 10% Fetal Bovine Serum (FBS) and Penicillin-Streptomycin (P/S) was added for the purpose of trypsin inactivation and the cells were suspended. After the cell suspension was filtrated through 100-μm and 40-μm cell strainers, the obtained cells were centrifuged, re-suspended in DMEM (10% FBS and P/S) and then plated on 15 cm2 plates.

To induce iPSC reprograming, MEFs were treated with 2 μg/mL Dox to induce 4F and then transferred to on irradiated MEFs as feeder cells at 3 days after Dox treatment. On the next day, the cells were cultured in ESC medium containing 10% FBS and Leukemia inhibitory Factor (LIF)-containing conditioned medium, which was obtained from the 293A cells stably transfected with pCAG-LIF-IRES-puro (LIF-expressing 293A cells).

### METHODS DETAILS

#### Tissue preparation and IHC

For paraffin-section, tissues were harvested, fixed in 10% neutralized formalin for 2 days and then stored in 70% ethanol until further processing. For frozen-section, tissues were harvested, fixed in 4% paraformaldehyde (PFA) for overnight, submerged with 30% sucrose and then embedded into OCT compound. H&E staining and IHC were performed following standard protocols. The following antibodies were used for IHC: anti-GFP (Abcam, 6673, 1:400; Clontech, JL-8, 1:200); anti-Klf4 (Cell signaling, 3728, 1:200); anti-Ki67 (Cell signaling, 12,202, 1:200); anti-Sox9 (Abcam, 185230, 1:100); anti-Alb (R&D, MAB1455, 1:200); anti-BrdU (Abcam, 6326, 1:100); Phalloidin-488 (ThermoFisher Scientific, A12379, 1:400). (ThermoFisher Scientific, A12379, 1:400). After staining, the sections were mounted by DAPI Fluoromount-G (SouthernBiotech, 100–20) for nuclear counterstain.

#### RNA isolation and quantitative-PCR (qPCR)

Total RNAs were isolated using TRIzol reagent (ThermoFisher) and RNeasy Mini kit (Qiagen) according to the manufacturer’s instructions. RNA samples were treated with RNase-Free DNase Set (Qiagen). RT was performed with Maxima Reverse Transcriptase (ThermoFisher) followed by qPCR using Platinum SYBR Green quantitative PCR supermix (ThermoFisher) in a thermocycler. The levels of expression of respective genes were normalized to corresponding Nat1 values. Primer sequences are listed in Table S1.

#### Bulk RNA-Sequencing

Isolated tissues were homogenized with a polytrone in Trizol. The extracted RNA was purified with RNeasy Mini Kit from the homogenates. RNA quality was assessed and all samples had a minimum RNA integrity number (RIN) of 8.6. RNA library preps were prepared using the Illumina TruSeq Stranded Total RNA Sample Prep kit with Ribo-zero Gold (cat. no. RS-122–2301). Briefly, RNA was depleted of ribosomal RNA and mitochondrial RNA, then fragmented and reverse transcribed. cDNA was end-repaired, adenylated, ligated with sequencing primers and PCR amplified. Libraries were pooled and sequenced on the HiSeq 4000 using paired-end 150 base-pair (bp) to a depth of 50 + million uniquely aligned reads per experiment. Reads were mapped to the mouse genome (mm10) using STAR (v2.5.3a PMID: 23104886) with default parameters. Gene expression levels were calculated using HOMER (v4.9.1 PMID: 20513432) by quantifying the uniquely aligned reads to the exons of RefSeq genes. Differential expression (DE) analysis was performed using R package DESeq2 (v1.22.2 https://genomebiology.biomedcentral.com/articles/10.1186/s13059-014-0550-8) using pairwise experiment design and genes with a false discovery rate (FDR) < 0.05 and absolute log fold-change (logFC) > 0.5 were identified as significantly different. Clustering was performed using Cluster 3.0 and Java TreeView. Venn Diagram was created using R package “VennDiagram” (https://cran.r-project.org/web/packages/VennDiagram/index.html).

#### ATAC-sequencing

All ATAC-seq analysis was performed on two biological replicates. Frozen liver samples were processed for ATAC-seq using the omni-ATAC seq protocol (Corces et al., 2017). Libraries were constructed using published Nextera PCR primers (Buenrostro et al., 2013) and checked for quantity on a TapeStation 2200 (Agilent). All sequencing was performed using pair-end 42bp reads on an Illumina NextSeq 500 sequencer. Adapter sequences were trimmed using TrimGalore (v0.4.5 http://www.bioinformatics.babraham.ac.uk/projects/trim_galore/).

Paired-end reads were aligned to the mm10 mouse genome using bwa mem (v0.7.12 PMID: 19451168). Alignment bam files were further processed by samtools (v1.9 PMID: 19505943) for quality filtering, duplicates removal, indexing, and sorting. For each sample, peaks were called by macs2 (v2.1.2 https://genomebiology.biomedcentral.com/articles/10.1186/gb-2008-9-9-r137). Small regions (<1000 bp) with local enriched signals passing the FDR cutoff 0.05 were called narrow peaks. Then the called narrow peaks from all samples were merged to get a union peak file. HOMER (v4.9.1 PMID: 20513432) was used to quantify the read counts in each of the union peak. Resulted read counts were normalized to total number of mapped reads by DESeq2 (v1.22.2 https://genomebiology.biomedcentral.com/articles/10.1186/s13059-014-0550-8) to identify the DE peaks with cutoff of FDR <0.05 and absolute logFC >1 called by HOMER getDiffExpression.pl.

Clustering of peak intensities were performed using k-means clustering with k = 6 after manual inspection of a range of k on the z-scaled log-transformed normalized peak intensities. Heatmap was generated using R package “gplots” (https://cran.r-project.org/web/packages/gplots/index.html). Motif enrichment analysis for each cluster of peaks was performed using HOMER (v4.9.1 PMID: 20513432).

Clustering of DE peak intensities were performed using k-means clustering with “Lloyd” algorithm at 1000 iteration and k = 6 after manual inspection of a range of k on the z-scaled log-transformed normalized peak intensities. Heatmap was generated using R package “gplots” (https://cran.r-project.org/web/packages/gplots/index.html) on 1000 sub-sampled peaks. Motif enrichment analysis for each cluster of peaks was performed using HOMER (v4.9.1 PMID: 20513432).

#### Principle component analysis (PCA)

Normalized gene expression count or peak count by DESeq2 “rlog” function was used for PCA. Top 500 most variable genes or peaks were analyzed by R function “prcomp”.

#### Pathway analysis

Pathway analysis was performed by WebGestalt (https://academic.oup.com/nar/article/47/W1/W199/5494758) using over-representation method on the Gene Ontology Biological Process Non-redundant database. Significant cutoff was FDR 0.05.

#### ScRNA-sequencing

For Single-Cell-RNA-Seq, Hep4F mice were treated with or without Dox (0.1 mg/mL) in drinking water for 1 day and Dox water was replaced by normal water. Liver samples were collected 1 day after Dox withdrawal and subjected to liver cell isolation. Liver cells were isolated after collagenase perfusion as described before (Cabral et al., 2018). Briefly, hepatocytes and other cells were isolated separately with different gravity of centrifuge and both fractions were mixed at the same number of cells. Then, these fractions were subjected to scRNA-Seq using Next GEM Single Cell 3^ˊ^ kit v3.1. Single-Cell reads were processed by 10X cell ranger pipeline (v3.1.0 https://support.10xgenomics.com/single-cell-gene-expression/software/pipelines/latest/what-is-cell-ranger) and then analyzed by R package “Seurat” (v3.0 https://www.cell.com/cell/fulltext/S0092-8674(19)30,559-8). In brief, cells with more than 45% of reads mapped to mitochondria or with less than 2000 reads were removed before the analysis. Then data was normalized and scaled using the default method. The top 2000 most variable genes and the first 20 correlated components were used as anchors to integrate the two samples. Uniform Manifold Approximation and Projection (UMAP) and clustering were performed using the top 20 principle components of the integrated data. Plots were generated by R package “ggplot2” (https://cran.r-project.org/web/packages/ggplot2/index.html).

#### Bisulfite sequencing

Bisulfite sequencing was performed as described previously (Takahashi et al., 2017). Briefly, bisulfite conversion was carried out using Zymo EZ DNA Methylation-Gold kit (Zymo Research) according to the manufacturer’s protocol, followed by PCR with Epi-Taq (Takara). Amplified fragments were subcloned using TOPO TA Cloning Kit for Sequencing (Thermo Fisher Scientific) and individually sequenced. Obtained sequences were analyzed by QUMA application (http://quma.cdb.riken.jp).

#### Comprehensive metabolic panel analyses

Hep-4F and Alb-Cre (Control) mice were treated with Dox for 2 days and then sera were collected, subjected to VETSCAN VS2 Chemistry Analyzer (Zoetis).

#### Western blotting

Liver nuclear extracts were prepared using NE-PER Nuclear and Cytoplasmic Extraction Reagents (Thermo Scientific) according to manufacturer’s instructions. Proteins were resolved using 4–12% Bis-Tris Nu-PAGE (Thermo Scientific), transferred to a polyvinylidene fluoride membrane, and probed using indicated primary antibodies conjugated with horseradish peroxidase. Specific protein bands were detected using ECL Plus Western Blotting Substrate (Thermo Fisher). Below antibodies were used: anti-TOP2A (1:1000, ab52934, Abcam); anti-TOP2B (1:1000, ab220385, Abcam); β-actin (1:2000, sc-47778, Santa Cruz Biotechnology).

#### Decatenation assay

The decatenase activity of Top II in liver nuclear extracts was measured via using the Human Topoisomerase II Assay Kit (kDNA Based) (TopoGEN), according to manufacturer’s instructions.

#### Top2a knockdown

Lentiviral vectors for small hairpin RNAs (shRNAs) against Top2a expression were generated by Gateway system as described previously. Briefly, a 19-base shRNA-coding fragment with a 5’-ACGTGTGCTGTCCGT-3′ loop was subcloned into pENTR-H1-tetO, subjected to Gateway LR reaction with pCS-RfA-ETHygro to obtain Dox-inducible shRNA lentiviral plasmids for Top2a. and a scrambled control were generated by subcloning the following oligonucleotides together with their complementary sequences into pCS2tetO-shRNA-ET2.

To produce viruses carrying a specific shRNA expression unit, lentiviral inducible shRNA plasmids were co-transfected with pCAG-HIVgp and pCMV-VSVG-RSV-Rev in 293FT cells. Lipofectamine 3000 (Invitrogen) was used for the transfection according to manufacturer’s instructions. Viral supernatants were collected around 48 h after transfection and passed through a 0.45 μm filter to remove cellular debris.

#### iPSC reprogramming

MEFs were isolated from reprogrammable mice (*Col1a1*^2loxP-tetO–4F/2loxP-tetO–4F^;*ROSA26*^rtTA/rtTA^) as described previously. For lentiviral infection, the MEFs were infected with prepared lentiviruses and cultured with 150 μg/mL hygromycin 2 days after infection. To induce iPSC reprograming, MEFs were cultured in the DMEM medium containing 10% Fetal Bovine Serum (FBS) with 2 μg/mL Dox to induce 4F and then transferred to on irradiated MEFs as feeder cells at 3 days after Dox treatment. On the next day, the cells were cultured in ESC medium containing 10% FBS and Leukemia inhibitory Factor.

### QUANTIFICATION AND STATISTICAL ANALYSIS

For comparisons, unpaired t test or one-way ANOVA with Tukey’s post hoc analysis were used with GraphPad Prism 8. Statistical details are indicated in the figure legends. Values with p < 0.05 are considered statistically significant.

## Supplementary Material

Supplement

## Figures and Tables

**Table:** KEY RESOURCES TABLE

REAGENT or RESOURCE	SOURCE	IDENTIFIER
Antibodies		

Goat polyclonal anti-GFP	Abcam	Cat# 6673; RRID: AB_305643
Mouse monoclonal anti-GFP	Clontech	Cat# JL-8; RRID: AB_10013427
Rabbit monoclonal anti-Ki67	Cell signaling	Cat# 12202; RRID: AB_2620142
Goat polyclonal anti-Klf4	R&D	Cat# AF3158; RRID: AB_2130245
Rat monoclonal anti-Sox9	Abcam	Cat# 185230; RRID: AB_2715497
Rat monoclonal anti-BrdU	Abcam	Cat# 6326; RRID: AB_305426
Rabbit monoclonal anti-Top2a	Abcam	Cat# 52934; RRID: AB_883143
Rabbit monoclonal anti-Top2b	Abcam	Cat# 220385; RRID: N/A (No longer available)
Mouse monoclonal anti-β-actin	Santa Cruz	Cat# 47778; RRID: AB_2714189
Mouse monoclonal anti-Afp	Santa Cruz	Cat# sc-8399; RRID: AB_626665
Rabbit monoclonal anti-GS	Abcam	Cat# 49873; RRID: AB_880241
Mouse monoclonal anti-E-cadherin	MBL	Cat# NCH-38; RRID: AB_10982676

Chemicals, peptides, and recombinant proteins		

Doxycycline	Sigma-Aldrich	Cat# D9891
DAPI Fluoromount-G	SouthernBiotech	Cat# 100–20
OCT compound	Fisher HealthCare	Cat# 4585
TRIzol	Invitrogen	Cat# 15596026
RNeasy Mini kit	QIAGEN	Cat# 74134
RNase-Free DNase Set	QIAGEN	Cat# 79254
Maxima H Minus Mastermix	Thermo Scientific	Cat# M1662
Platinum SYBR Green quantitative PCR supermix	Bio Rad	Cat# 1725274
Carbon tetrachloride	Sigma-Aldrich	Cat# 289116
Zymo EZ DNA Methylation-Gold kit	Zymo Research	Cat# D5005
Epi-Taq	TaKaRa	Cat# R110A
ICRF-193	Enzo Life Sciences	Cat# BML-GR332
Dexrazoxane hydrochloride	MedChemExpress	Cat# HY-76201
4-Acetamidophenol, 98%	Thermo Scientific	Cat# 102332500
NE-PER^™^ Nuclear and Cytoplasmic Extraction Reagents	Thermo Fisher Scientific	Cat# 78833
NuPAGE^™^ 4 to 12%, Bis-Tris, 1.0 mm, Mini Protein Gel, 10-well	Thermo Fisher Scientific	Cat# NP0321BOX
Immobilon-P Membrane, PVDF	Millipore	Cat# IPVH00010
SuperSignal^™^ West Femto Maximum Sensitivity Substrate	Thermo Fisher Scientific	Cat# 34094
DMEM	Thermo Fisher Scientific	Cat# 11995
Penicillin-Streptomycin	Thermo Fisher Scientific	Cat# 15140122
FBS	Gemini Bio-products	Cat# 25300054
Trypsin-EDTA (0.05%)	ThermoFisher Scientific	Cat# 15140122
Lipofectamine3000	Invitrogen	Cat# L3000015
Hygromycin B	Invitrogen	Cat# 10687010
Polybrene	Sigma	Cat# H9268

Critical commercial assays		

Human Topoisomerase II Assay Kit	TopoGEN	Cat# TG1001-2
Infinity^™^ ALT (GPT) Liquid Stable Reagent	ThermoFisher	Cat# TR71121

Deposited data		

RNA-Seq, ATAC-Seq, sc-RNA-Seq	This paper	GSE144600

Experimental models: Cell lines		

Reprogrammable MEFs	This paper	N/A
293FT cells	Salk Stem cell core	N/A
LIF-expressing 293A cells	This paper	N/A

Experimental models: Organisms/strains		

Mouse: B6N.Cg-*Speer6-ps1*^*Tg(Alb-cre)21Mgn*^/J	The Jackson Laboratory	RRID: IMSR_JAX:018961
Mouse: *Gt(ROSA)26Sor*^tm1(rtTA*M2)Jae^ *Col1a1*^*tm3(tetO-Pou5f1, −Sox2, −Klf4, −Myc)Jae*^/J	The Jackson Laboratory	RRID: IMSR_JAX:011004
Mouse: FVB/N-Tg(tetO-MYC)36aBop/J	The Jackson Laboratory	RRID: IMSR_JAX:019376
Mouse: B6.Cg-*Gt(ROSA)26Sor*^*tm1(rtTA, EGFP)Nagy*^/J	The Jackson Laboratory	RRID: IMSR_JAX:005,670
Mouse: *Gt(ROSA)26Sor*^tm1(rtTA*M2)Jae^ *Col1a1*^*tm4(tetO-Pou5f1,-Sox2,-Klf4,-Myc)Jae*^/J	The Jackson Laboratory	RRID: IMSR_JAX:011,011

Oligonucleotides		

See Table S1 for qPCR primers	N/A	N/A
See Table S2 for bisulfite primers	N/A	N/A
See Table S3 for Top2a target	N/A	N/A

Recombinant DNA		

pCAG-LIF-Ires-Puro	This paper	N/A
pCS-tetO-shTop2a-EThygro	This paper	N/A
pCS-tetO-shLuc-EThygro	This paper	N/A
pCAG-HIVgp	Miyoshi. H et al., 1998	Cat# RDB04394
pCMV-VSVG-RSV-Rev	Miyoshi. H et al., 1998	Cat# RDB04393

Software and algorithms		

Prism 8.0	GraphPad Software	http://www.graphpad.com
ImageJ	Software	https://imagej.nih.gov/ij/
Excel	Microsoft	N/A

## Highlights

- Hepatocyte-specific 4F expression induces cell proliferation and dedifferentiation
- Hepatocyte-specific 4F expression induces a global change in DNA accessibility
- Top2a is required for cellular reprogramming *in vitro* and *in vivo*
- *In vivo* reprogramming has beneficial effects on regenerative capacity
