# Supplementary material for: In vivo partial cellular reprogramming enhances liver plasticity and regeneration
Source: Cell Rep. Author manuscript; Available in PMC 2023 Jan 2. (PMC9807246; doi:10.1016/j.celrep.2022.110730)
Supplement: Supplement [file NIHMS1845386-supplement-Supplement.pdf]

**Cell Reports, Volume *39***

## Supplemental information

# *In vivo* partial cellular reprogramming enhances liver plasticity and regeneration

**Tomoaki Hishida, Mako Yamamoto, Yuriko Hishida-Nozaki, Changwei Shao, Ling Huang, Chao Wang, Kensaku Shojima, Yuan Xue, Yuqing Hang, Maxim Shokhirev, Sebastian Memczak, Sanjeeb Kumar Sahu, Fumiyuki Hatanaka, Ruben Rabadan Ros, Matthew B. Maxwell, Jasmine Chavez, Yanjiao Shao, Hsin-Kai Liao, Paloma Martinez-Redondo, Isabel Guillen-Guillen, Reyna Hernandez-Benitez, Concepcion Rodriguez Esteban, Jing Qu, Michael C. Holmes, Fei Yi, Raymond D. Hickey, Pedro Guillen Garcia, Estrella Nuñez Delicado, Antoni Castells, Josep M. Campistol, Yang Yu, Diana C. Hargreaves, Akihiro Asai, Pradeep Reddy, Guang-Hui Liu, and Juan Carlos Izpisua Belmonte**

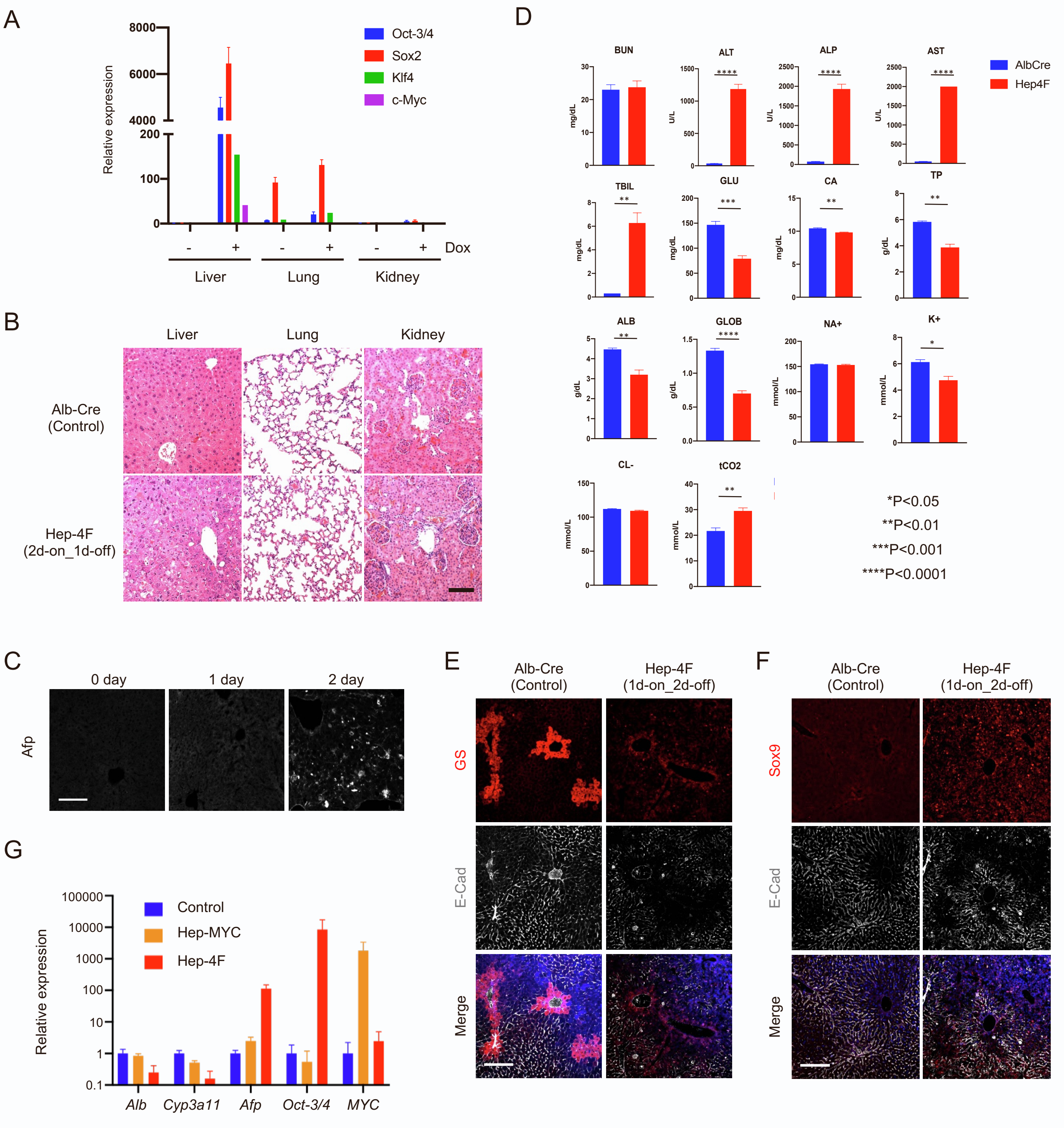


**Figure S1. Liver-specific 4F induction, Related to Figure 1.**

**(A)** qPCR analysis for 4F expression in the liver, lung and kidney of Hep-4F mice treated with or without Dox for 2 days. Data represent the mean with SE (n=3, biological replicates). **(B)** Livers collected from Dox-treated and untreated Hep-4F mice 2 days after Dox administration. Scale bar, 100 mm. **(C)** Afp protein expression in Hep-4F mice. IHC for Afp in the livers collected from Hep4F treated with Dox. Scale bar, 100 mm. **(D)** Comprehensive metabolic panel analyses of reprogramming livers. Alb-Cre or Hep-4F mice were treated with Dox for 2 days and then serum samples were collected, subjected to metabolic panel analyses. Data represent the mean with SE (Alb-Cre mice, n = 3; Hep-4F mice, n =4, biological replicates). **(E)** Impact of 4F on liver zonation. IHCs were performed for indicated proteins on liver sections prepared from Alb-Cre mice (control) and Hep-4F mice treated with Dox. Scale bar, 200 mm. **(F)** Sox9 distribution in reprogramming livers. IHCs for Sox9 and E-cad on liver sections prepared from Alb-Cre mice (control) and Hep-4F mice treated with Dox. Scale bar, 200 mm. **(G)** Effects of MYC and 4F on hepatic markers. Hep-Myc and Hep-4F mice were treated with Dox for 1 day and RNA samples were isolated from the livers collected from the mice 1 day after Dox withdrawal (1d-on_1d-off). These RNAs were subjected to qPCR. Data represent the mean with SE (n=3, biological replicates).

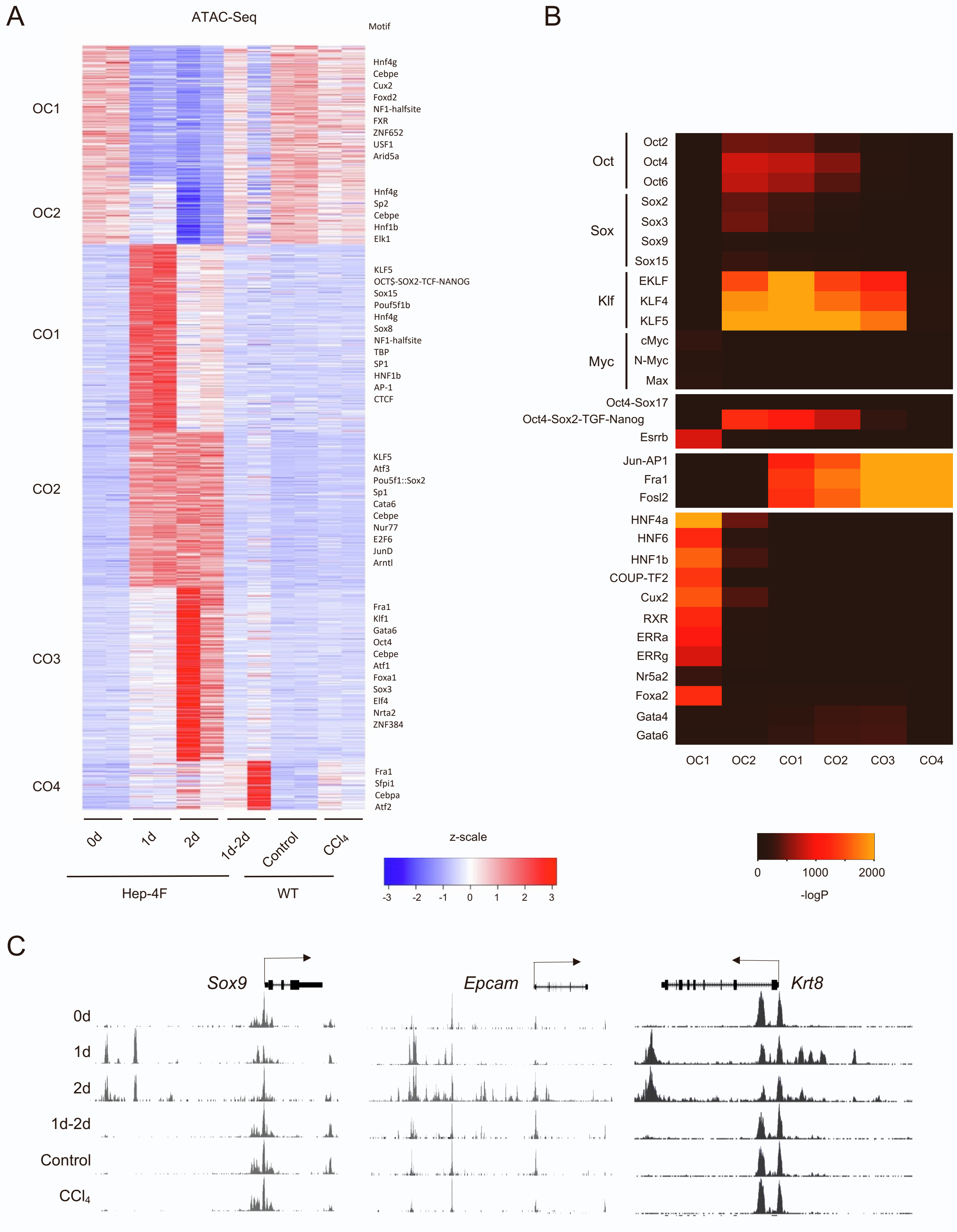


**Figure S2. Analysis of ATAC-Seq data, Related to Figure 2.**
**(A)** Clustering analysis for ATAC-Seq peaks, which produced 6 clusters, depending on the time when the loci changed from open to closed (OC) or open to closed (CO). Enriched motifs are shown in the right of the heatmap. **(B)** TF motif enrichment analyses. **(C)** Genome browser view of the ATAC-Seq data at the liver progenitor markers loci.

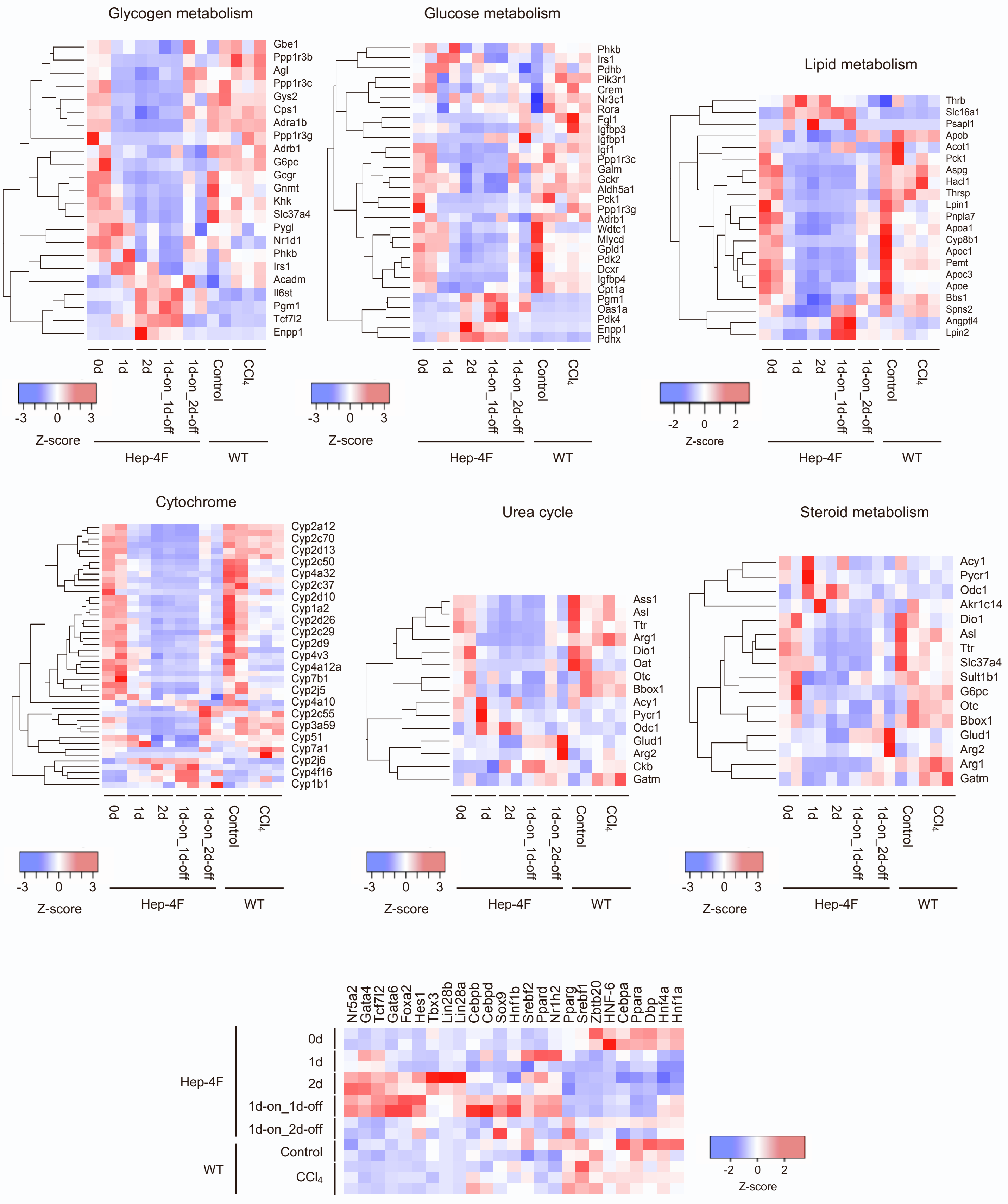


**Figure S3. Heatmap of liver signature in Hep-4F mice, Related to Figure 2.**
Heatmaps were generated from RNA-Seq, related to Figure 2E.

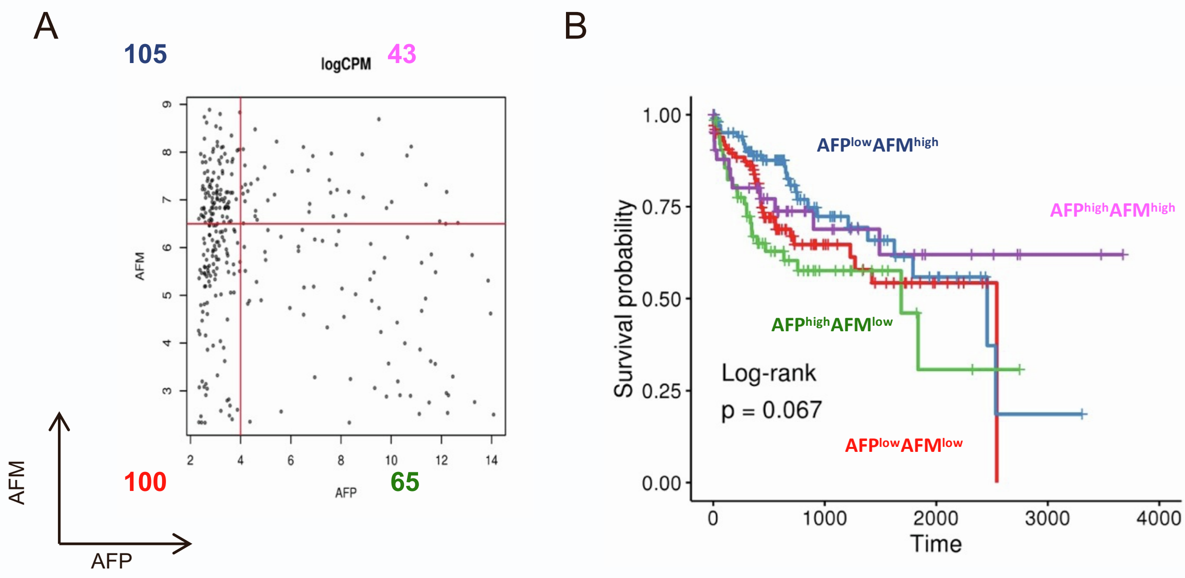


**Figure S4. Analyses of public data set of HCCs, Related to Figure 2.**
**(A)** Scatter plot of AFP and AFM. **(B)** Survival rate of each group.

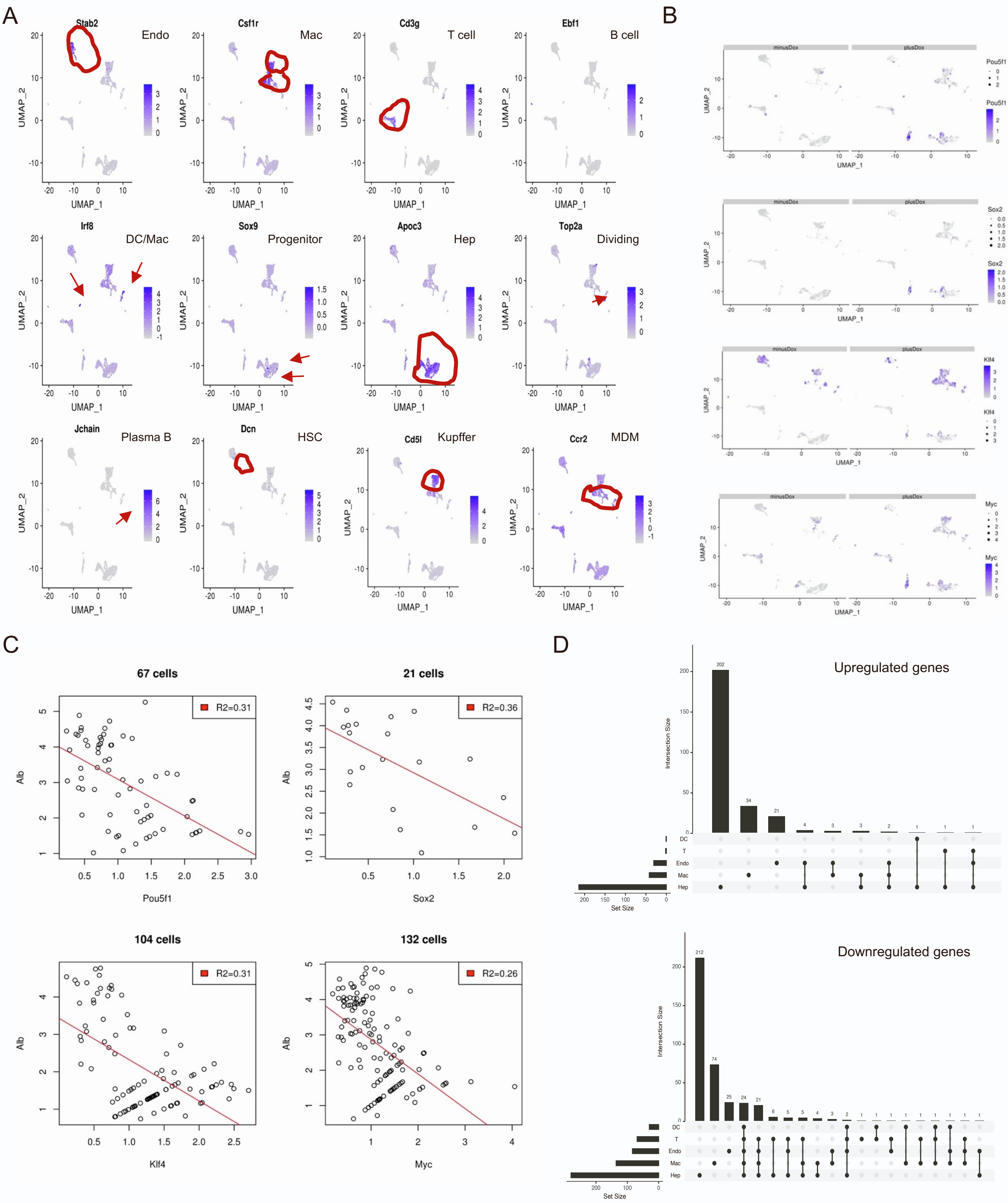


**Figure S5. Analysis of scRNA-Seq data, Related to Figure 4.**
**(A)** Characterization of each cell-type based on gene expression of cell-type specific markers. **(B)** UMAP visualization for 4F expression levels. **(C)** Correlation of gene expression levels of each 4F gene and Alb levels. **(D)** DEG analyses within each cluster in scRNA-Seq.

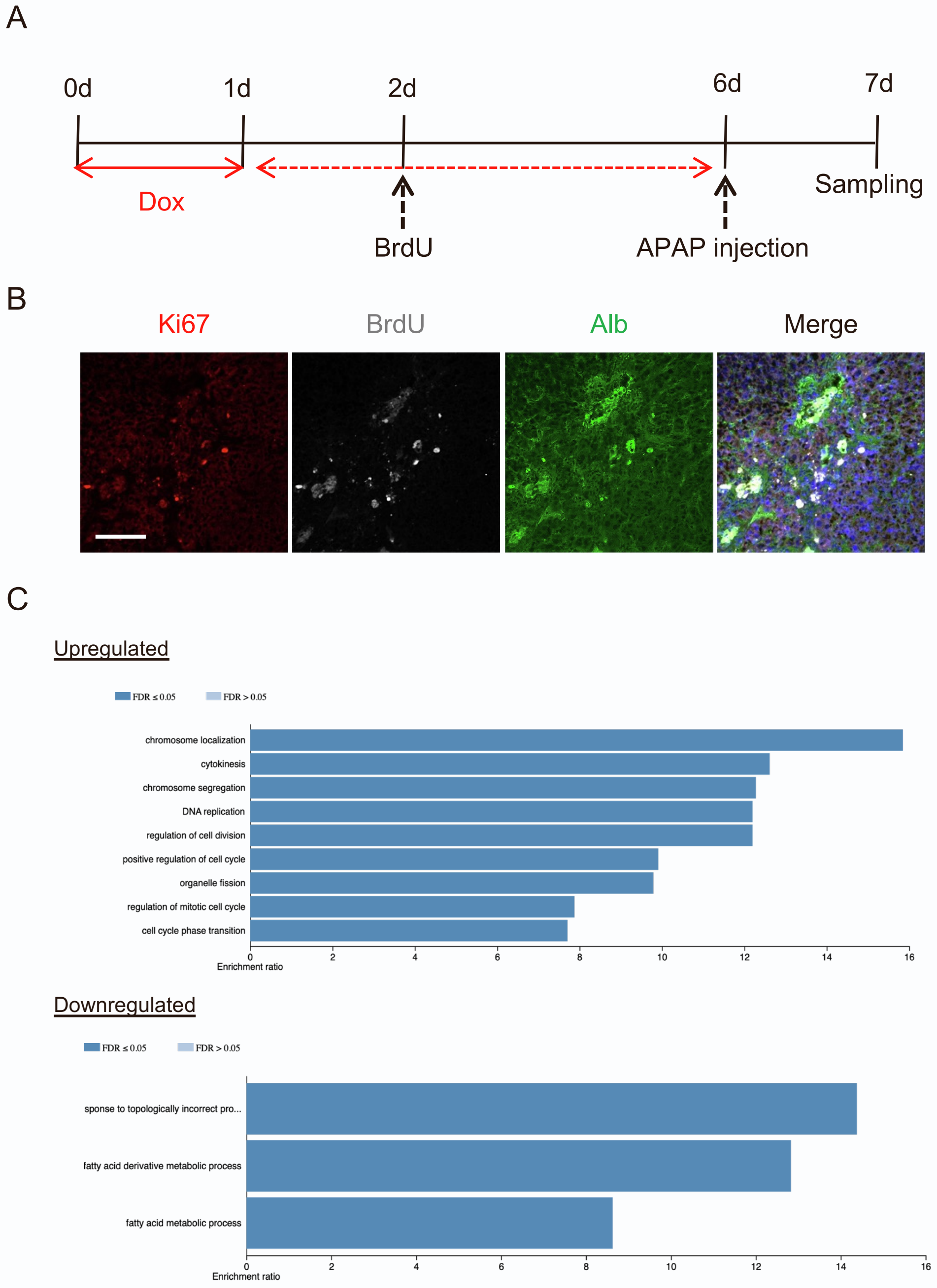


**Figure S6. Analysis of partially-reprogrammed livers after liver injury, Related to Figure 6.** **(A)** Schematic representation for APAP treatment protocol for cell tracking of BrdU-positive cells. **(B)** IHC for Ki67, BrdU and Alb. Scale bar, 100 mm. **(C)** GO analyses of DEGs before and after liver injury in Hep-4F.

**Table S1. Primers for qPCR, Related to STAR Methods.**

| Gene | Sense | Antisense |
|---|---|---|
| Nat1 | ATTCTTCGTTGTCAAGCCGCCAAAGTGGAG | AGTTGTTTGCTGCGGAGTTGTCATCTCGTC |
| Oct-3/4 | GGCTTCAGACTTCGCCTTCT | TGGAAGCTTAGCCAGGTTCG |
| Sox2 | TTTGTCCGAGACCGAGAAGC | CTCCGGGAAGCGTGTACTTA |
| Klf4 | GCACACCTGCGAACTCACAC | CCGTCCCAGTCACAGTGGTAA |
| Alb | TCCTGATTGCCTTTTCCCAGTATCT | GCCAGTTCACCATAGTTTTCACGGA |
| Cyp3a11 | GACAAACAAGCAGGGATGGAC | CCAAGCTGATTGCTAGGAGCA |
| Afp | TCCCTCATCCTCCTGCTA | GCACATTCTTCTCCGTCAC |
| Cited1 | AGGGTCTCCGGGATCTGTC | TCTAGCCCCAATTCAACCACC |
| Hnf1a | AGGAGTGTAATAGGGCGGAGT | GAGGTCCGTTATAGGTGTCCA |
| Hnf1b | CCCCTCACCATCAGCCAAG | GGTTCTGAGATTGCTGGGGATT |
| Hnf4a | CTACGGAGCCTCGAGCTGT | CCACACATTGTCGGCTAAAC |
| Hnf6 | AGACCTTCCGGAGGATGTG | TGGACGTCTGTGAAGACCAG |
| Cebpa | TGGACAAGAACAGCAACGAGTAC | GCAGTTGCCCATGGCCTTGAC |
| Cebpb | GGTTTCGGGACTTGATGCA | CAACAACCCCGCAGGAAC |
| Cebpd | CCCCAAAGCTATGTGCCTTTC | CCTGGAGGGTTTGTGTTTTCTG |
| Foxa2 | CATGGGACCTCACCTGAGTC | CATCGAGTTCATGTTGGCGTA |
| Gata4 | CGAGGGTGAGCCTGTATGTAA | CTGCTGTGCCCATAGTGAGAT |
| Gata6 | GACTGTCCTGTGCCAACTGTCA | TGGAGTTTCATATAGAGCCCGC |
| Sox9 | CCACGGAACAGACTCACATCTCTC | AGGAAGCTGGCAGACCAGTA |
| Nanog | CCTTGGAATGCTGCTCCGCTC | CCCAGCAACCACATGGTGGCT |
| MYC (Tg) | ACCAGATCCCGGAGTTGGAA | CGTCGTTTCCGCAACAAGTC |

**Table S2. Primers for bisulfite sequencing, Related to STAR Methods.**

| Target | Sense | Antisense |
|---|---|---|
| Dppa3 promoter-1 | TTTTTTTATTTTGTGATTAGGGTTGGG | CTTCACCTAAACTACACCTTTAAAC |
| Dppa3 promoter-2 | TTTGTTTTAGTTTTTTTGGAATTGG | CTTCACCTAAACTACACCTTTAAAC |
| Oct-3/4 distal enhancer | TTTAGGTTTTAGAGGTTGGTTTTG | CCAATTTCTATACATTCATTATAAAACAAT |

**Table S3. shRNA target sequence, Related to STAR Methods.**

| Target | Sequence |
|---|---|
| Top2a | GGTGAAGTTTAAGGCCCAAGTAG |
| Luc | CGTACGCGGAATACTTCGA |
